# Optimizing Telepractice Selection and Implementation for Persons with Aphasia

**DOI:** 10.5195/ijt.2024.6604

**Published:** 2024-06-28

**Authors:** Elizabeth O. Tibus, Maryanne Weatherill, Amy D. Rodriguez

**Affiliations:** 1 Center for Visual and Neurocognitive Rehabilitation, Joseph Maxwell Cleland Atlanta VA Medical Center, Decatur, Georgia, USA; 2 Department of Neurology, Emory School of Medicine, Emory University, Atlanta, Georgia, USA

**Keywords:** Aphasia, Aphasia-friendly, Healthcare, Telepractice, Telerehabilitation, Teleresearch

## Abstract

Telepractice is used to conduct many aspects of healthcare, including rehabilitation and research. However, information regarding how to identify optimal candidates and overcome barriers to participating in telepractice are limited. In the context of aphasia rehabilitation research, we developed two tools for optimizing telepractice: (1) the Participant Technology Questionnaire (PTQ), an aphasia-friendly tool for gathering information about potential telepractice participants; and (2) the Virtual-Appropriate Decision Approach (VADA), a framework for assessing and modifying methods that support virtual activities. The PTQ provides valuable information about the effects of human, technology and setting influences that may impact the success of transitioning activities to a virtual format, while the VADA takes findings from the PTQ one step further into application. The PTQ and the VADA can help researchers and clinicians with planning and directing virtual engagement, and both tools have potential to be applied broadly in all areas of telepractice.

The growth of tele-based services including telehealth, telemedicine, telepractice, and telerehabilitation to connect providers and researchers with their clients, patients, and participants since the onset of the COVID-19 pandemic has forever changed the landscape of healthcare and rehabilitation. Our lab focuses on post-stroke aphasia rehabilitation research and consists of speech-language pathologists who are members of The American Speech Language Hearing Association (ASHA). Given that ASHA prefers the term telepractice to avoid excluding non-healthcare settings from its use, telepractice is the term we will use throughout this paper. ASHA defines telepractice as “the delivery of services using telecommunication and internet technology to remotely connect clinicians to clients, other health care providers, and/or educational professionals for screening, assessment, intervention, consultation, and/or education” (ASHA, n.d.-c).

The rise of telepractice has highlighted users' challenges associated with this service delivery mode such as lacking computer experience, internet access issues, or age-related considerations. Nearly two-thirds of adults over the age of 70 have hearing loss (Lin et al., 2011), and the loss of perception and cognition associated with normal aging can influence their ability to successfully participate in telepractice (Nieman & Oh, 2020). In 2018, 13 million Americans over age 65 were not ready to participate in tele-activities largely due to inexperience with technology and 6.8 million were reportedly unready due to limitations such as dementia, poor hearing, reduced vision, and communication impairments (Lam et al., 2020). Seniors in lower socioeconomic groups are less likely to have reliable internet access, basic computer experience, or caregiver support (Jezewski et al., 2022). Older persons may have limited experience with online meetings or may not own or have access to equipment necessary compensate for sensory losses, such as headphones, a microphone, or a large computer monitor. Attitudes towards technology may dissuade individuals from meeting via videoconferencing while others may be open to connecting via virtual means. Low computer anxiety, perceived security, physician input, and self-efficacy with using the technology are predictors of success with telepractice in adults ages 55-75 (Cimperman et al., 2013). These factors that contribute to the success or failure of telepractice should be considered when identifying potential clients and study participants.

Individuals with stroke-induced aphasia, whom we refer to as persons with aphasia, or PWA, are a unique demographic and may experience more difficulties connecting online than adults who have never experienced a stroke due to their communication impairments. This may include difficulty following written or verbal directions or verbalizing needs when connection difficulties arise. In 2020, 11.7 million people globally experienced stroke (Tsao et al., 2022) and millions of people worldwide are living with aphasia (National Aphasia Association Aphasia Statistics, 2023; ASHA, n.d.-a). Post-stroke aphasia occurs after initial ischemic strokes in 15% of adults ages 65 and younger and increases to 43% of stroke patients aged 85 and older (ASHA, n.d.-a). After our in-person aphasia research activities were forced to pause, we pivoted to conducting some of our research activities remotely. Because it is the responsibility of the speech-language pathologist to choose appropriate clients/participants for telepractice (ASHA, n.d.-c), it was necessary to investigate whether the PWA enrolled in our aphasia treatment study could adapt to using videoconferencing platforms to continue their participation. To ensure data integrity, we needed a resource to screen participants' readiness to perform research activities before resuming our studies using only technology-based modalities. Given PWA's and the aging population's potential challenges using technology and the perpetuity of telehealth, a tool to address all variables that could impact telepractice engagement was needed. It was prudent to investigate sensory abilities, views on technology, and connectability and synthesize this information to determine readiness to take part in online tasks.

## Purpose of the Study

The purpose of the study was to identify the best method for shifting our research activities halted by the COVID-19 pandemic from in-person to online while maintaining participant engagement and data integrity. We had three goals: (1) locate and utilize a screening tool capable of identifying research participants likely to successfully complete research activities remotely; (2) identify and mitigate challenges to telepractice for PWA using the tool; and (3) apply the information gathered by the tool to a problem-solving process for decision-making and implementation.

## Methods

### Creation of the PTQ—The Search for Available Tools

In June 2020, we began to seek out and evaluate currently available technology questionnaires. First, we queried local sources in the fields of rehabilitation and assistive technology for tools and then reviewed available tools to determine if the content addressed target areas for successful participation in virtual assessment and treatment for research. We started in the most intuitive places—searching the ASHA website, asking fellow speech-language pathologists and researchers in the Atlanta area about protocols in use, and emailing experts in assistive technology (AT) and geriatrics at several U.S. universities for an already existing tool.

Our search yielded limited results. None of our local colleagues had an available protocol or screening tool that fulfilled all our needs. “Considerations for Speech Language and Cognitive Assessments Via Telepractice” posted on the ASHA website (ASHA, n.d.-b) provided information about getting started in telepractice, choosing clients and appropriate assessments, licensure requirements, policies, documentation, reimbursement, coding, and cultural, linguistic, and ethical considerations. The Emory Telehealth Technology Access and Skills Survey (Hajjar et al, 2020) asked about access to technology, use, comfort, and skill level using electronics at home but did not provide detailed information concerning users' environment, troubleshooting abilities, support system, hearing and vision, or confidence and attitudes toward participating in online research activities. Emory University's Technology Attitudes and Efficacy Questionnaire (Vickers, n.d.) addressed users' feelings and comfort level towards technology but lacked basic information about users' access to required computer hardware, software, and experience with technology use. Out of the five specialists in AT and geriatrics contacted, one replied and gave suggestions for online assessments that were emerging but did not know of any comprehensive technology-readiness questionnaires.

### Creation of the PTQ—Search Results

Following initial inquiries to ASHA and local colleagues, a detailed internet search was conducted in June 2020. Search terms included combinations of keywords “identifying” “successful” “technology use” “aphasia” “older adults” “geriatric” “seniors” “candidates” “technology adoption” “computer” “internet” “computer readiness questionnaire,” “technology questionnaire,” “computer skills questionnaire,” “computer competence assessment,” and “computer readiness checklist.” Search results yielded four entities that provided resources related to technology use: Tools for Life, Georgia's Assistive Technology Act program, improved access to and acquisition of AT services and devices and also offered AT assessments, training, resources, and funding information to individuals with disabilities in Georgia; Vanderbilt University's Center for Rehabilitation Engineering and Assistive Technology studied and designed assistive technologies while training engineers in biomechanics and related fields; The Collaborations in Health, Aging, Research and Technology (CHART) at the University of Illinois promoted successful aging through developing technology, education, and research; Pearson Assessments' “Hello Examinee” checklist for clients gave suggestions for preparing the environment, lighting, supplies, computer settings, and setting up a separate camera (Pearson Education Inc., 2020). These assistive technology-related resources matched adults with disabilities with AT devices, described biomechanical devices and prostheses, provided audio and visual support for older adults, and listed steps for clients to take to prepare for their virtual appointment but did not present a useful telepractice-readiness screening tool.

Internet search results also yielded a few technology-related questionnaires. The TechSAge Minimum Battery (Gonzalez et al, 2020), a 168-item self-reporting questionnaire developed by the Rehabilitation Engineering Research Center on Technologies (TechSAge RERC), surveyed older adults' demographics, health, sensory abilities, tech experience, and memory deficits with the goal developing a database that identifies technology needs among older adults with vision, hearing, and mobility impairments. The Center for Research and Education on Aging and Technology Enhancement (CREATE) at Cornell University's Computer Proficiency Questionnaire (CPQ) asked about users' ability to perform computer tasks using a 5-point Likert scale in six different areas: computer basics, printer, communication, internet, calendar, and entertainment (Boot et al., 2015). CREATE's Technology Experience Profile (TEP) measured frequency of using six different areas of technology: communication technology, computer technology, everyday technology, health technology, recreational technology, and transportation technology (Barg-Walkow et al., 2014). The Media and Technology Usage and Attitudes Scale (MTUAS) quantified frequency of technology use over 44 items and attitudes towards technology over 12 items (Rosen et al., 2013). The Telehealth Usability Questionnaire (TUQ) inquired about usefulness of telehealth systems including ease of use, interface quality, reliability, satisfaction, and quality of the interaction (Parmanto et al., 2016) while the Telemedicine Patient Questionnaire (TMPQ) queried patients' views on risks and benefits of telecare (Demiris et al., 2000). The Telemedicine Satisfaction and Usefulness Questionnaire (TSUQ) and the Telemedicine Satisfaction Questionnaire (TSQ) investigated patient satisfaction with telemedicine (Bakken et al., 2006; Yip et al., 2003), the Technology Acceptance Model (TAM) identified the relationships between telehealth system design features, patients' perception of usefulness, ease, and attitudes and their actual usage of the systems (Davis, 1993), and the Post-Study System Usability Questionnaire (PSSUQ) probed user satisfaction with usability of computer systems (Lewis, 1995).

The available questionnaires focused on telehealth and telemedicine usability, system design, users' perceived benefit of use, ease of use, patient satisfaction, frequency of use, and users' proficiency with basic computer skills. However, none of the questionnaires retrieved via community resources or by internet search supplied a cohesive profile of a user's environment, computer skills and accessories, sensory abilities, or comfort, confidence, and attitudes towards technology in a format favorable to persons with communication disorders. Additionally, none of the available questionnaires offered guidance or answers to our questions: How can we identify individuals with aphasia who can successfully participate in, and complete research tasks conducted remotely? How will we assess the effectiveness of our newly implemented procedures for conducting online language assessments and other research activities and modify them if needed? The lack of available resources to assist with answering these questions prompted us to create two tools for optimizing telepractice: the Participant Technology Questionnaire (PTQ), an aphasia-friendly, 8-page questionnaire and the Virtual-Appropriate Decision Approach (VADA), a problem-solving process developed for assessing and modifying telepractice for research participation.

### Creation of the PTQ—Content

After carefully reviewing existing resources and their shortfalls, we determined seven necessary components of the PTQ: Living Situation, e.g., type of environment and persons who may be in the home; Sensory Abilities, e.g., hearing and vision abilities; Equipment, e.g., access to type of equipment required for video conferencing; Internet Use, e.g., internet access and ability to troubleshoot connection problems; Comfort, e.g., comfort level and ability using household technology; Confidence, e.g., indicating confidence level for completing study activities online; and Attitudes Towards Technology, e.g., attitudes related to the impact of technology on society. These seven components give the research team valuable information about variables that may affect a participant's competence and willingness to complete research tasks conducted using a virtual meeting platform. The PTQ also provides information to make adjustments that may be needed for all stages of the virtual meeting. Having this information ahead of time not only increases the likelihood of successful completion of telepractice activities but also helps ensure the integrity of the data acquired during the session. Table 1 represents the topics included in the PTQ and existing technology questionnaires in 2020 demonstrating that the PTQ is the only tool that covers all seven areas.

**Table 1:** Technology-related Questionnaires and PTQ Components

Instrument	Living situation	Internet use	Equipment	Sensory abilities	Comfort	Confidence	Attitudes
Emory TTASS		X	X		X		
Emory TAQ					X		X
RERC TechSAge	X	X		X			
CPQ		X					
TEP		X					
MTUAS		X					X
TUQ					X		X
TMPQ					X		X
TSUQ							X
TSQ					X		X
TAM							X
PSSUQ					X		X

*Note.* Emory TTASS= Emory Telehealth Technology Access and Skills Survey; Emory TAQ= Emory Technology Attitudes and Efficacy Questionnaire; RERC TechSAge= Rehabilitation Engineering Research Center on Technologies to Support Aging among People with Long-Term Disabilities; CPQ= Computer Proficiency Questionnaire; TEP= Technology Experience Profile; MTUAS= Media and Technology Usage and Attitudes Scale; TUQ= Telehealth Usability Questionnaire; TMPQ= Telemedicine Patient Questionnaire; TSUQ= Telemedicine Satisfaction and Usefulness Questionnaire; TSQ= Telemedicine Satisfaction Questionnaire; TAM= Technology Acceptance Model; PSSUQ= Post-Study System Usability Questionnaire.

### Creation of the PTQ—Aphasia-Friendly Formatting

Because our participants have aphasia, the PTQ was written according to aphasia-friendly principles (Rose et al., 2012) comprising sans serif clear font with important text bolded, 1.5 and double spacing, numbers written as figures, and blank spaces around the text with one exception: we used 12-point font rather than 14-point font to allow clear divisions between the sections of the questionnaire and to avoid overlapping of the sections from one page to the next. The questionnaire also used simplified language, shortened phrases, and avoided double negatives to aid with comprehension.

### Application of the PTQ—Use with Research Participants

Immediately after our lab restarted operations, the PTQ was given to two participants who served as pilots to help us “tweak” the questionnaire items and create operating procedures for participants with aphasia. One pilot participant had hearing loss (M, age 75) while the other pilot participant (F, age 82) had hearing loss, mild cognitive impairment, and limited computer experience. After modifications were made to the PTQ based on use with our two pilot participants, we began using the tool with our aphasia research participants. To date, we have administered the PTQ to 18 participants (14 male, 4 female, age 23-83 years old), with chronic aphasia due to left-hemisphere stroke (6-112 months post-onset). PWA were recruited into our aphasia rehabilitation studies using methods approved by institutional review boards at Emory University and the Atlanta VA Medical Center (#116506, #102356, #277). Written informed consent was obtained from participants prior to implementation of any study procedures. One of the 18 participants was already enrolled in the research study, so the PTQ was utilized to gauge their potential continuation in research activities. For the remaining 17 participants, the PTQ was part of the study onboarding process. It was mailed to the participant along with consent forms, completed by either the participant or their caregiver, and returned to the study team via mail. Responses were reviewed by the study team ahead of the consenting appointment, allowing the researcher to prepare and make necessary adjustments before the first online session.

### The Development of the Virtual-Appropriate Decision Approach (VADA)

**Figure 1:**
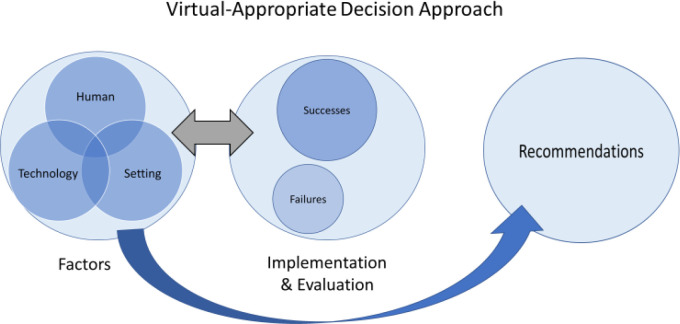
The VADA Model

To create a framework for successfully transitioning research activities to a virtual format using information obtained by the PTQ, we further synthesized the seven areas into three main factors: human, setting, and technology influences. Thus, the PTQ subsequently informed the development of the VADA, a systematic problem-solving process for application in telepractice. The VADA is supported by an illustrative figure and application schematic. (See Figure 1.) The VADA addresses research implementation, refining and enhancing concepts related to the Plan-Do-Study-Act (PDSA) cycle which are traditionally used as performance improvement tests of change (Reed & Card, 2016) and which are necessary to incorporate the differing goals of human subjects research (Mormer & Stevans, 2019). While traditional models of clinical quality improvement such as PDSA are frequently used for healthcare processes, differences exist between human subjects research and quality improvement practice (Mormer & Stevans, 2019). Using the VADA and the PTQ, information was gathered, processes were investigated, developed, and planned (Plan) then carried out by the research team (Do). While the tasks were being conducted, there was simultaneous as well as retrospective analysis of success/failure (Study), which resulted in immediate revisions (Act). By identifying and defining relevant human, technology, and setting factors, the VADA targets elements that influence the “Do” aspect of telepractice, while continuing to incorporate important aspects of reflection and revision.

The VADA model is comprised of three components: (1) factors; (2) implementation and evaluation; and (3) recommendations. *Factors* represent potential strengths and weaknesses in human, setting, and technology factors identified in the PTQ and their effects on the feasibility for telepractice. See Table 2 for a description of how the VADA factors and elements of the PTQ overlap.

**Table 2:** The VADA Factors and How They Relate to the Seven Components of the PTQ

	Human	Setting	Technology
Living Situation	X	X	
Sensory Abilities	X		
Equipment		X	X
Internet Use	X	X	X
Comfort	X		
Confidence	X		
Attitudes	X		

The human components of the VADA are analyzed as they relate to the clinician's attitudes, skills, and constraints as well as participants' specific cognitive, motor, and other personal factors (e.g., sensory abilities, confidence, motivation) that may influence participation. Does a person's aphasia limit their ability to read or follow directions? Is the person anxious about using the telepractice platform? It is known that participant characteristics (ASHA, n.d.-c) and impairments users experience pose additional challenges using telepractice and should be considered (Lathan et al., 1999); however, knowing the person's strengths can help us to capitalize on those abilities to promote overall success. The setting considers the participant's environment, safety, and security, as well as privacy policies of the clinician's institution that may act as constraints on the telepractice session. Access to software and hardware for conducting telepractice sessions are primary technology considerations. In addition to their separate effects, these three factors are also interconnected and cannot be fully addressed separately. Based on the combination of human, setting and technology factors, a particular technique may be developed for implementation and evaluation. *Implementation and evaluation* of techniques reveal whether they are successful or unsuccessful. If a technique is not successful, the factors are re-evaluated, a new technique is developed, and the implementation and evaluation process is repeated. Once techniques and strategies are refined through an iterative process, they become recommendations. *Recommendations* shape the individualized methods that can be carried out with a participant, as needed.

## Results

Our goals were to: (1) locate and utilize a screening tool capable of identifying research participants likely to successfully complete research activities remotely; (2) identify and mitigate challenges to telepractice for PWA using information gathered from the PTQ; (3) apply the information through the VADA problem solving process for decision-making and implementation.

Goal #1: We failed to identify a comprehensive screening tool. Neither our local resources nor our internet search produced a comprehensive tool to determine our participants' readiness for online research activities. Thus, we created the PTQ for use in telepractice.

Goal #2: Twenty participants completed the PTQ. Sections 1-4 of the PTQ asked about Living Situation, Sensory Abilities, Equipment, and Internet Use. Most participants (85%) lived with spouse or family in a private home/apartment/condominium (90%). Eighty percent relied on glasses or contact lenses to correct their vision and 20% of these participants had difficulty seeing even with corrective lenses. Five participants reported wearing bilateral hearing aids with 3 of the 5 reporting hearing difficulty despite amplification. Participants reported being more tech-savvy than expected. Most participants (90%) had an internet connection and had met with a healthcare professional via Zoom or other virtual meeting format. Most (85%) had access to a computer with a webcam and headphones (60%). About half (55%) had downloaded a new app or software in the previous 6 months and half of participants said they had access to a printer. These responses helped the research staff anticipate and respond to needs to make the virtual meeting run as effectively as possible and ensure integrity of the data being collected.

In Section 5, Comfort, most participants reported ability and comfort using household technologies (cell phone, tablet, computer, webcam, mouse, online meeting format) either independently or with help. The level of comfort using a webcam was the lowest (67%) largely because of lack of experience, while comfort using a cell phone was the highest at 94%. Similarly, most participants expressed a high level of assurance using technology in Section 6, Confidence. Half or more of respondents said they were “very confident” doing 9 of the 12 tasks listed. They reported being least confident in 3 areas: (1) their ability to use their computer without stress, fatigue, or discomfort; (2) their ability to modify computer settings if needed; (3) their ability to troubleshoot internet connection problems or setting difficulties.

Responses targeting Attitudes Towards Technology in Section 7 were mixed. Half of the views regarding technology were favorable (life is better with technology, I am comfortable using technology, advancing technology has had a positive impact on society); the other half were unfavorable (overreliance on technology, technology causing stress, technology complicating life). More than one respondent chose “not sure” as their answer to 4 out of the 6 items probed, thereby skewing an already small sample size. See tables 3,4,5 and 6 in the Appendices for detailed participant responses.

**Figure 2:**
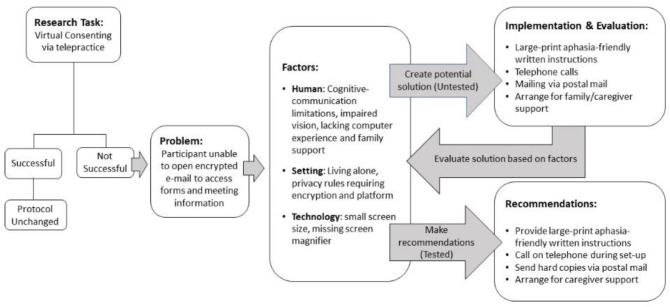
The VADA Flowchart for Telepractice

Goal #3: Potential challenges to completing online consenting and language assessments were identified using data from the PTQ and run through the VADA process. Modifications were implemented to maximize the likelihood of a valid outcome. An example of utilization is Mr. Q., a 78-year-old PWA who lives alone and has vision impairment and cognitive-communication deficits post-stroke. The Task is to complete the consenting process via a web-based meeting platform with Mr. Q. Facility policies require all emails be encrypted. Hard copies of aphasia-friendly instructions for opening encrypted emails were mailed via U.S. Postal Service to Mr. Q. ahead of his appointment. The Problem is Mr. Q. has difficulty opening the encrypted email containing the handouts and video link for the session. Information from the PTQ is gathered and Factors are analyzed: Human factors include his cognitive and visual deficits, minimal computer experience, and lack of family assistance; Setting factors include his solo living situation and privacy rules of the research institution; Technology factors include small computer screen size and lacking screen magnifier. Next, potential solutions are tested and modified (Implementation and Evaluation) and Recommendations are made for how to optimize the telepractice experience. Figure 2 exemplifies VADA utilization in research telepractice.

## Discussion

There is no doubt that clinical and research service delivery methods across many professions have changed as a result of the COVID-19 pandemic. Because of the variety of professions and populations with varying skill levels using telepractice, in our case aphasia researchers and PWA, we identified a need at the start of the pandemic for a tool that can identify potential challenges people with aphasia may face when participating in virtual assessment. This was important to mitigate the frustrations PWA may already experience while communicating and to ensure valid and effective interactions. Although questionnaires investigating consumers' competency using telehealth (Taylor & Little, 2023) and older adults' digital literacy (Choi et al., 2023; Scherrenberg et al., 2023) have since emerged, most questionnaires related to users' experiences with tele-based health services have focused on usability and satisfaction with the system being used (Hajesmaeel-Gohari & Bahaadinbeigy, 2021). No single resource was found in 2020 that offered a clear picture of participants' access to technology, skill level and experience with computers and associated devices, troubleshooting abilities, physical space, visual and hearing acuity, confidence, comfort, and attitudes towards technology, and help identify special accommodations necessary for successful virtual participation. To fill this gap, we created the PTQ, a comprehensive, user-friendly tool that affords researchers and clinicians valuable information about the effects of human, technology and setting influences that potentially impact the success telepractice activities. The information gathered from the PTQ can then be applied to the VADA, a problem-solving schematic useful for tailoring the online session to ensure a positive experience for researchers and clinicians as well as their participants and clients.

These necessary and useful tools for optimizing telepractice are not without limitations. The PTQ relies on participants' self-reporting or perceived ability levels, which may or may not be accurate. Additionally, the PTQ has been utilized with a relatively small sample size of PWA and responses on particular items are limited due to modifications made during the tool's evolution. Thus, we have not yet established approaches to barriers and facilitators based on specific response profiles, but this may be possible in the future. Finally, clinicians and researchers may perceive the absence of a scoring system as a limitation; however, we believe a score is less useful and informative than the qualitative information provided by the PTQ, which reveals the specific component(s) requiring modification(s) unique to an individual.

## Future Directions

With continued use of the PTQ and the VADA, it may be possible to identify specific patterns of responses that align with approaches that will help optimize telepractice. Because the factors that either help or hinder successful telepractice are relevant to all rehabilitation disciplines, we believe the PTQ and VADA may be easily translated to a variety of clinical and research settings.

## Conclusion

The growth of tele-based service delivery has necessitated exploration of human, setting and technology factors relevant to clients and participants to facilitate successful telepractice. We sought a tool that could provide information about participants' readiness to engage in research tasks conducted remotely. To fill the void of an existing screening tool and framework for optimizing telepractice with clients and participants, we created the PTQ and the VADA.

## Appendix A Summary of Participant Responses to the PTQ Sections 1-4

**Table:**

Internet Use					
Have a home internet connection?	Browser used?	Able to troubleshoot internet difficulties?	Is there someone else in the home who can troubleshoot?	Ever seen a health professional via video call?	Which virtual meeting format was used?
No n=2 (10%)	Chrome n=13 (65%)	No n=9 (45%)	No n=6 (30%)	No n=3 (15%)	Zoom n=14 (70%)
Yes n=18 (90%)	Safari n=1 (5%)	Yes n=10 (50%)	Yes n=6 (30%)	Yes n=17 (85%)	WebEx n=1 (5%)
	Edge n=2 (10%)	NR n=1 (5%)	NA n=8 (40%)		VAVC n=1 (5%)
	NA n=2 (10%)				NA n=3 (15%)
	NR n=2 (10%)				NR n=1 (5%)

Environment		Sensory Abilities			
Living Situation?	Type of housing?	Wear glasses or contact lenses?	Trouble seeing even when wearing glasses?	Wear a hearing aid?	Trouble hearing, even with hearing aids?
Alone n=3 (15%)	Relative's home n=2 (10%)	No n=4 (20%)	No n=16 (80%)	No n=15 (75%)	No n=16 (80%)
With spouse/family n=17 (85%)	House/apt/condo n=18 (90%)	Yes, for distance n=4 (20%)	Yes n=4 (20%)	Yes, both ears n=5 (25%)	Yes n=3 (15%)
Other n=0 (0%)	Assisted Living Facility n=0 (0%)	Yes, to see up close n=3 (15%)	NA n=0 (0%)	Yes, right ear n=0 (0%)	NR n=1 (5%)
	Independent Senior Facility n=0 (0%)	Yes, bifocals n=9 (45%)		Yes, left ear n=0 (0%)	NA n=0 (0%)

Equipment				
Have or can borrow a computer?	Mac or PC?	Desktop or Laptop?	Own a tablet?	Device connected to a printer?
No n=3 (15%)	Mac n=3 (15%)	Desktop n=4 (20%)	No n=10 (50%)	No n=6 (30%)
Yes n=17 (85%)	PC n=11 (55%)	Laptop n=9 (45%)	Yes n=8 (40%)	Yes n=10 (50%)
	NR n=3 (15%)	Both n=2 (10%)	NA n=2 (10%)	NA n=4 (20%)
	NA n=3 (15%)	NA n=3 (15%)		
		NR n=2 (10%)		

Which OS used?	Have a webcam?	Own earbuds/headphones?	If yes, do you regularly use earbuds/headphones?	Last download of new app or software?
Apple n=2 (10%)	No n=3 (15%)	No n=6 (30%)	No n=11 (55%)	Never n=4 (20%)
Windows n=13 (65%)	Yes n=12 (60%)	Yes n=12 (60%)	Yes n=1 (5%)	In last 6 months n=11 (55%)
Not sure n=1 (5%)	NA n=5 (15%)	NA n=2 (10%)	NA n=8 (40%)	In the last year n=1 (5%)
NA n=3 (15%)				I don't know n=4 (20%)
NR n=1 (5%)				

*Note.* NR = No Response; NA = Not Applicable; VAVC = Veterans' Administration Video Connect; OS = Operating System

## Appendix B Participants' PTQ Responses Regarding Level of Comfort (PTQ Section 5)

**Table:**

HOW COMFORTABLE ARE YOU USING THESE ITEMS?	I'm not sure, I've never used	I can't use, even with help	I can use, but need help	I can use, no help needed	NA	NR
Cell phone	1	0	8	9	2	
iPad/other tablet	3	2	7	6	2	
Laptop computer	4	0	6	8	2	
Desktop computer	2	0	7	8	2	1
Webcam	6	0	6	6	2	
Computer mouse	2	0	6	10	2	
Zoom/Teams/other online meeting format	1	1	8	8	2	

*Note.* NA = Not Applicable; NR = No Response. Two participants were given an older version of the form which asked about frequency of use instead of comfort.

## Appendix C Participants' PTQ Responses Regarding Confidence Using Technology (PTQ Section 6)

**Table:**

Indicate your level of confidence regarding the following statements:				
	Not Confident	Somewhat Confident	Very Confident	NR
I have the capabilities and stamina to use electronic devices (computer or tablet) without stress, fatigue, or discomfort.	5	5	9	1
I have a quiet environment free from distractions that will allow me to focus on my electronic device.	1	6	12	1
I am able to see my computer or tablet screen clearly.	2	5	12	1
I am able to clearly hear sounds coming from my tablet or computer with or without external speakers.	2	5	12	1
I have the physical capabilities to move a computer mouse.	2	5	12	1
I can follow directions to modify my electronic device settings if instructed.	3	7	8	2
I am able to focus on my screen for an hour or more.	4	5	10	1
I have reliable internet access.	2	4	13	1
I am able to troubleshoot connection or setting difficulties I may encounter when using my computer or tablet.	7	6	6	1
Communication and personal information I share via tablet or computer will be confidential.	1	4	14	1
I will be able to complete all study tasks using my tablet or computer.	3	6	10	1
I will benefit from participating in this research study.	1	4	14	1

*Note.* NR = No Response. One participant did not fill out this section.

## Appendix D Participants' PTQ Responses Regarding Attitudes Towards Technology (PTQ Section 7)

**Table:**

Indicate your level of agreement with the following statements about technology:						
	STRONGLY DISAGREE	DISAGREE	NOT SURE	AGREE	STRONGLY AGREE	NA
Life is better with technology. The more tech the better!	1	2	5	10	2	
I am comfortable using technology.	1	4	4	8	3	
People rely too much on technology these days.	1	6	3	10		
Technology causes me stress. I just can't keep up!	2	5	4	6	3	
Advancing technology has had a positive impact on society.			1	9	2	8
Technology makes life more complicated.	1	1	1	1	4	12

*Note.* NA = Not Applicable. Eight participants responded to the statement: “Technology makes life more complicated.” Twelve participants responded to the statement: “Advancing technology has had a positive impact on society.”
